# Pathogenesis and Treatment of Thrombohemorrhagic Diathesis in Acute Promyelocytic Leukemia

**DOI:** 10.4084/MJHID.2011.068

**Published:** 2011-12-21

**Authors:** Anna Falanga, Laura Russo, Carmen J Tartari

**Affiliations:** Division of Immunohematology and Transfusion Medicine, Dept. Oncology-Hematology, Ospedali Riuniti, Bergamo, Italy

## Abstract

Acute promyelocytic leukemia (APL) is a distinct subtype of myeloid leukemia characterized by t(15;17) chromosomal translocation, which involves the retinoic acid receptor-alpha (RAR-alpha). APL typically presents with a life-threatening hemorrhagic diathesis. Before the introduction of all-trans retinoic acid (ATRA) for the cure of APL, fatal hemorrhages due, at least in part, to the APL-associated coagulopathy, were a major cause of induction remission failure. The laboratory abnormalities of blood coagulation found in these patients indicate the occurrence of a hypercoagulable state. Major determinants of the coagulopathy of APL are endogenous factors expressed by the leukemic cells, including procoagulant factors, fibrinolytic proteins, and non-specific proteolytic enzymes. In addition, these cells have an increased capacity to adhere to the vascular endothelium, and to secrete inflammatory cytokines [i.e. interleukin-1beta (IL-1beta) and tumor necrosis factor (TNF-alpha)], which in turn stimulate the expression of prothrombotic activities by endothelial cells and leukocytes. ATRA can interfere with each of the principal hemostatic properties of the leukemic cell, thus reducing the APL cell procoagulant potential, in parallel to the induction of cellular differentiation. This effect occurs *in vivo*, in the bone marrow of APL patients receiving ATRA, and is associated with the improvement of the bleeding symptoms. Therapy with arsenic trioxide (ATO) also beneficially affects coagulation in APL. However, early deaths from bleeding still remain a major problem in APL and further research is required in this field. In this review, we will summarize our current knowledge of the pathogenesis of the APL-associated coagulopathy and will overview the therapeutic approaches for the management of this complication.

## Introduction

Thrombo-hemorrhagic complications in patients with hematologic malignancies are as frequent as in those with solid tumors and significantly affect morbidity and mortality.^1^ In acute leukemias, thrombosis and bleeding manifestations may occur concomitantly as a part of the same thrombo-hemorrhagic syndrome (THS).^2^–^4^ Furthermore, a hypercoagulable state is present in virtually all of these patients, even without clinical manifestations. The pathogenesis of hypercoagulability is complex, and a central role is played by the fundamental molecular changes of the leukemic cells, which overexpress procoagulant factors, as well as adhesion molecules and cytokines capable of inducing procoagulant changes in the vascular wall and of stimulating multiple cellular interactions. Recent molecular studies in experimental models of human tumors have demonstrated for the first time that oncogene and repressor gene-mediated neoplastic transformation induces activation of blood coagulation. Similarly, in cells from patients with acute promyelocytic leukemia (APL) the t(15;17) chromosomal translocation induces hyperexpression of tissue factor (TF) and renders the patient hypercoagulable.^5^ Patients with acute leukemias very often present with a range of laboratory abnormalities consistent with the diagnosis of disseminated intravascular coagulation (DIC) and a variety of clinical manifestations, ranging from localized venous or arterial thrombosis to diffuse life-threatening bleeding. The incidence of these complications varies according to the type of leukemia and to the phase of treatment. In patients with APL hemorrhage is usually predominant,^6^ and worsens during induction chemotherapy when large volumes of tumor cells are being destroyed rapidly. The presence of the coagulopathy is a significant risk factor for early hemorrhagic death in APL patients, and remains the most important cause of death during the induction therapy in these patients.^7^

In recent years, DIC complicating the presentation of APL has received new interest, due to several advances in translational research, including: (1) enhanced understanding of the biology of APL; (2) greater sensitivity of diagnostic tests for subclinical DIC; and (3) development of new therapies for remission induction, including all-trans-retinoic acid (ATRA) and Arsenic Trioxide (ATO). ATRA promotes the terminal differentiation of leukemic promyelocytes and ATRA-induced remission of APL is accompanied by the prompt improvement of the coagulopathy of this disease.^8^ ATO also induces the molecular remission of APL and a simultaneous rapid resolution of the related coagulopathy. The combination therapy of ATRA and ATO is effective in inducing APL remission in newly diagnosed patients, and may provide an alternative to ATRA+chemotherapy in this disease, with less toxic effects.^9^ One mechanism of ATRA effect on the coagulopathy relies on its capacity to reduce the APL cell procoagulant activities (PCA) [i.e. tissue factor (TF) and cancer procoagulant (CP)]. Less information is available on the effects of ATO or the combination ATO+ATRA on the PCA of APL cells. In this review we will focus on the pathogenesis and proposed treatment of the THS occurring in APL, with particular attention to the effects of ATRA and ATO on the hemostatic system.

## The Clinical Features of the Coagulopathy of APL

APL is a distinct subtype of acute myeloid leukemia (AML-M3), which typically presents with a life-threatening hemorrhagic diathesis, the clinical and laboratory features of which are consistent with DIC.^2^,^10^ The bleeding disorder is particularly severe in the microgranular variant of APL (M3v), which is characterized by marked hyperleukocytosis.^11^–^12^ Before the introduction of ATRA, APL was characterized by high incidence of hemorrhagic death, due to intracranial and pulmonary hemorrhage, which significantly contributed to failure of remission induction.^13^ In a retrospective multicentre study of 268 consecutive APL patients, the overall remission rate was 62% and the prevalence of hemorrhagic deaths during induction therapy was 14%. No significant difference in the remission rate was observed in the groups of patients that received heparin, antifibrinolytic drugs or supportive therapy alone for controlling the coagulopathy.^14^ The use of ATRA for remission induction of APL has produced a complete remission rate >90%, together with a reduction of early hemorrhagic deaths to 2.4–6.5%.^4^,^15^ ATRA may however increase the risk for thrombosis in those patients with APL who manifest accelerated differentiation, otherwise known as the retinoic acid syndrome (RAS).^8^

Thrombosis and bleeding manifestations may occur concomitantly as a part of the same THS.^3^,^16^ Abnormalities of the blood clotting system are consistent with the diagnosis of DIC and are observed in the majority of these patients. Severe DIC with life-threatening bleeding involves the very rapid consumption of coagulation factors and platelets in the circulation as a consequence of the massive activation of intravascular clotting. Thrombocytopenia, caused by the replacement of bone marrow megakaryocytes by leukemic cells and by the hypoplasia subsequent to traditional chemotherapy, is aggravated by the consumption of platelets during clot formation. Secondary bacterial or viral infections in these susceptible hosts may further complicate the pathogenesis of the thrombocytopenia through a direct toxic effects, the generation of additional stimuli for activation of blood coagulation (e.g. bacterial endotoxin, interleukins, etc.).

In spite of a net increase in the rate of remission induction in patients with APL and overall improvement in survival, hemorrhage remains the most common cause of induction-related death in patients with APL, accounting for about 5% of cases in two consecutive PETHEMA (Programa Español de Tratamiento de las Hemopatias Malignas) group studies – PETHEMA LPA96 and LPA99.^7^ In these studies, the results of a multivariate analysis to identify the pre-treatment characteristics predictive of fatal hemorrhage found a significant role for the elevated white blood cell count (WBC >10 × 10^9^/L; P<0.0001) and the abnormal creatinine level (P<0.0004). In addition, the routine use of tranexamic prophylaxis (100mg/kg/d by continuous infusion) in the second study, failed to alter the risk of hemorrhagic death, while was associated with a trend toward a statistically significant increase in the thrombosis rate (6% vs. 3% in the LPA99 and LPA96 trial, respectively; P = 0.08 in multivariate analysis). Hemorrhagic mortality was almost exclusively due to intracranial and pulmonary hemorrhages. Fatal hemorrhagic events occurred from day 1 to day 23 with the majority noted in the first week; no lethal hemorrhages were documented beyond the fourth week of therapy.

A recent retrospective analysis of 771 consecutive patients with non-promyelocytic AML admitted to a single institution over the past 30 years revealed a similar risk for hemorrhagic death (55/771; 7%) but with improved results over the more recent past (e.g. 16% in the era 1977–1986 vs. 3% since 1994).^17^ Multivariate analysis identified the following factors predicting for hemorrhagic mortality in the AML patients: (1) WBC >50 × 10^9^/L, P<0.0001; (2) age > 60 years, P=0.04; (3) de novo AML, P = 0.005.^17^

Finally, the same PETHEMA database that was used for assessing hemorrhagic risk was subjected to a retrospective analysis for thrombosis risk in 759 consecutive APL patients.^7^ An incidence rate of thrombosis of 5.1% (39/759) was observed, and as noted above, 4 cases were associated with the use of tranexamic acid – 2 cases of deep vein thrombosis, 1 case of hemorrhagic skin necrosis and 1 of renal necrosis. In multivariate analysis, hypofibrinogenemia at presentation (<170 mg/dl) and the M3-variant subtype remained from the univariate analysis as independent prognostic factors. Thrombosis was observed to relate to a higher induction mortality (including deaths prior to the initiation of chemotherapy; 28% vs. 11%, P<0.01).^7^

## Hemostatic Laboratory Abnormalities Of APL Patients

**Figure 1:**
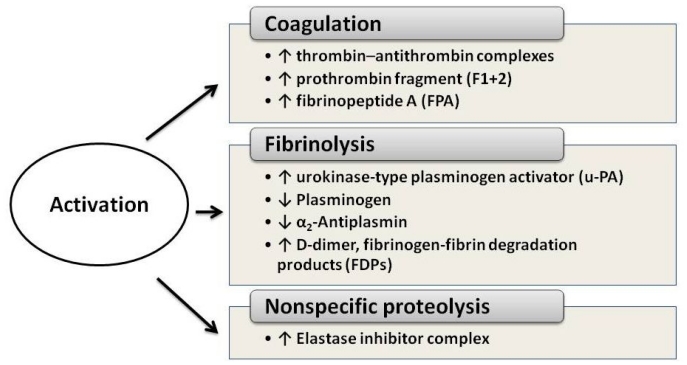
Laboratories abnormalities of hemostasis in APL patients.

In APL patients the most common abnormalities of routine clotting tests are: hypofibrinogenemia, increased circulating levels of fibrinogen-fibrin degradation products (FDPs), prolonged prothrombin and thrombin time. These abnormalities are not typical of any specific coagulation defect, but reflect the interaction of different pathophysiological processes, as similar alterations of routine clotting tests may derive from the activation of either coagulation, fibrinolysis and non-specific proteolysis These abnormalities can be accentuated by the initiation of cytotoxic chemotherapy, resulting in severe hemorrhagic complications.^18^ The use of new and more sensitive laboratory assays for the detection of coagulation products or enzyme inhibitor complexes confirms the activation of coagulation, fibrinolysis and non-specific proteolysis. As summarized in figure 1, plasma levels of markers of clotting activation, i.e. the prothrombin fragment 1+2 (F1+2), thrombin–antithrombin complexes (TAT) and fibrinopeptide A (FPA), are elevated in the majority of patients with APL.^2^,^19^–^20^ At the same time, high levels of FDPs and urokinase-type plasminogen activator (u-PA) together with low levels of plasminogen and alpha 2-antiplasmin are described and provide evidence for ongoing hyperfibrinolysis.^21^–^23^ Finally, plasma levels of leukocyte elastase and fibrinogen split products of elastase are also increased and testify to the elaboration of non-specific proteases.^24^ Activation of each of the three cascades (i.e. coagulation, fibrinolysis or non-specific protease) can potentially trigger the bleeding complications of APL. However, the new laboratory tests for the detection of markers of hypercoagulation demonstrate definitively that thrombin generation and fibrin formation are constant events in these patients. Of particular interest is the detection of elevated levels of D-dimer, the lysis product of stabilized cross-linked fibrin. This finding provides strong evidence that the hyperfibrinolysis typical of patients with APL is most likely secondary to the activation of the clotting system.^24^–^28^

Some studies have suggested that primary hyperfibrinolysis may be the major event leading to the bleeding diathesis in APL.^29^–^31^ However, on the basis of the laboratory tests currently available, it is difficult to prove the existence of primary hyperfibrinolysis in APL and even more difficult to establish the role of excessive fibrinolysis in triggering severe hemorrhage. In fact, while reactive, or secondary, hyperfibrinolysis, in response to clotting activation, can be easily documented in patients with leukemia, there are no specific tests that define primary hyperfibrino(geno)lysis *in vivo*. The findings of profound reductions of alpha 2-antiplasmin and plasminogen levels, which can be corrected with the therapeutic use of antifibrinolytic agents,^29^–^30^ do not allow the distinction between primary and secondary hyperfibrinolysis. Tapiovaara et al.^32^ observed that cells freshly isolated from the bone marrow of patients with APL expressed both urokinase (uPA) and tissue-type plasminogen activator (tPA). Findings of Mennell and colleagues^31^ showed that Annexin II, a protein with high affinity for plasminogen and for tPA, is highly expressed by leukemic cells isolated from APL patients, when compared to non-APL leukemic cells. They hypothesized that ‘dysregulated expression of annexin II on the surface of circulating APL cells’ could be responsible for primary hyperfibrinolysis *in vivo*. However, these authors assessed the activation of the fibrinolytic system in their patients with non-specific tests and demonstrated, among other abnormalities, elevated D-dimer levels in 8/10 patients. It is important noticing that treatment with ATRA is associated with an improvement in coagulation parameters, (i.e. F1+2, TAT, FPA, and D-dimer), as well as with a decrease in the plasma fibrinolytic potential. Indeed it iduces the synthesis of plasminogen activator (PA) inhibitors and inhibits the synthesis of annexin II, which leads to the downregulation of the receptor-bound PA activity.^24^–^27^ Finally, among other effects, ATRA also regulates the levels of von Willebrand factor (vWF).^33^

The beneficial effects of ATRA on parameters of coagulation, fibrinolysis and proteolysis activation are associated with improvement in clinical signs of the coagulopathy in the same patients. The benefits persists when ATRA is given in combination with chemotherapy.^24^,^33^–^34^ A study by Tallman et al.^34^ confirms the long-term benefit of induction therapy with ATRA on both disease-free and overall survival in APL and reaffirms the negative prognostic finding of clinical bleeding at the time of presentation.

## Pathogenesis of the Coagulopathy of APL

Consideration of the pathophysiology of the hypercoagulable state of APL patients is critical to the design of appropriate measures for intevention. Very recent molecular studies of experimental models of human cancer demonstrate that oncogene and repressor gene-mediated neoplastic transformation (e.g. activation of Met, loss of PTEN, induction of K-ras and loss of p53) activate clotting as an integral feature of neoplastic transformation.^5^ Triggering signaling pathways by one or more of these genes result in activation of blood coagulation and platelet function and/or suppression of fibrinolysis, which in some cases can produce thrombosis and/or DIC in these models.^35^–^37^ Similar signaling pathways have been predicted to play a similar role in APL. In these cells, the t(15;17) translocation, which results in the fusion of the nuclear retinoic acid receptor (RAR-alpha) gene on chromosome 17 with part of the PML (promyelocytic leukemia) gene on chromosome 15, induces hyperexpression of TF, again linking the primary oncogenic event with induction of hypercoagulability.^8^

**Figure 2:**
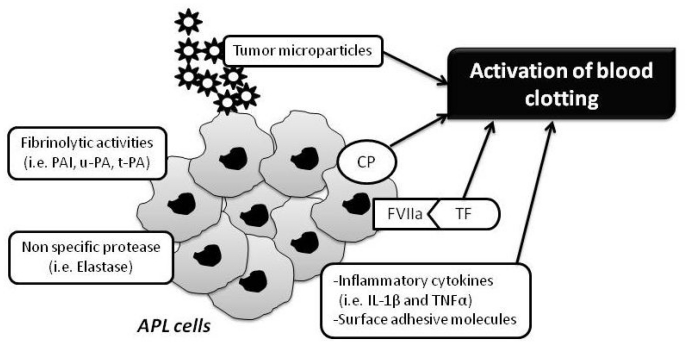
Procoagulant mechanisms of APL cells.

Although many exogenous factors, including cytotoxic chemotherapy and concomitant infections, can impair the normal delicate balance between procoagulant and anticoagulant forces in the hemostatic system of patients with APL, however the major determinants of coagulopathy in patients with APL are endogenous factors related to properties of the malignant leukemic cells and their interactions with host defense mechanisms. These properties include (Figure 2):

1. the expression of procoagulant activities, fibrinolytic proteins, and proteolytic enzymes,
2. the release of circulating microparticles,
3. the secretion of inflammatory cytokines,
4. the expression of surface adhesion molecules.

### 1. Procoagulant, fibrinolytic, and proteolytic properties

Leukemic cells isolated from APL patients and the NB4 cell line, the first human APL line containing the typical t(15;17) chromosomal balanced translocation, express high levels of procoagulant activity (PCA), including TF and cancer procoagulant (CP). They also provide an efficient alternative to platelets as a phospholipid surface for the assembly of the prothrombinase complex.

TF has been characterized in APL cells by several researchers.^38^–^41^ Others have demonstrated that CP also is expressed in leukemic blasts of various phenotypes and is found at the highest levels in patients with APL.^42^ ATRA-induced APL cell differentiation *in vitro* is associated with loss of the capacity to express either CP^43^ or TF.^44^–^45^ Further, both procoagulants are progressively reduced *in vivo* in the bone marrow cells of APL patients given ATRA for remission-induction therapy.^24^ Reduction of leukemic cell PCA by ATRA appears to be one important mechanism involved in the resolution of the coagulopathy. An *in vitro* study demonstrated that, after ATRA treatment, CP activity is down-regulated only in those NB4 cells that are sensitive to ATRA-induced cyto-differentiation, and not in ATRA-resistant cells that do not differentiate. However, TF activity was significantly reduced in all cell lines in response to ATRA, regardless of sensitivity to ATRA-induced differentiation.^46^ TF expression can be down-regulated by ATRA in both APL cells and in other types of leukemic cells^47^ and also in normally differentiated cells.^48^–^51^ Nuclear run-on experiments in human monocytes and monocytic leukemia cells support the concept that ATRA inhibits induction of TF expression at the level of transcription,^50^ but independently of the common transcription factors AP-1 or NF-kB.^50^ Zhu et al. demonstrated destabilization of TF mRNA induced by ATRA in NB4 cells, partially dependent upon protein synthesis,^51^ and Raelson and colleagues showed that ATRA induces synthesis of a protein in NB4 cells that selectively degrades PML/RAR-alpha fusion protein.^52^ Therefore, one or more proteins induced by ATRA in leukemic cells may also destabilize TF mRNA.^53^ Furthermore, this group reported that bone marrow cells from mice transgenic for the fusion genes PLZF-RAR-alpha or NPM-RAR-alpha express the TF gene, whereas the cells derived from those mice without the fusion gene do not express the TF gene.^54^ These data link directly, for the first time, the regulation of TF gene expression in APL cells with the malignant transforming events and provide strong support for the hypothesis that down-regulation of TF gene expression is a direct result of the mechanism of the ATRA effect on oncogene expression.

ATO, another agent effective in the cure of APL, including the APL resistant to ATRA also reduces TF expression and PCA of APL blast cells *in vitro* and *in vivo*.^55^ ATO exerts dose-dependent dual effects on APL cells: at low concentrations (0.5 μM), ATO induces partial differentiation by degrading the PML/RAR-alpha fusion protein; while at relatively high concentrations (0.5–2.0 μM), it triggers apoptosis.^56^ Zhou et al. recently published the evidence that ATO treatment can induce rapid loss of membrane procoagulant activity and TF mRNA leading beneficial effect on the related coagulopathy in APL.^57^–^58^ However, mechanisms by which ATRA or ATO lead to the rapid resolution of coagulopathy need further definition.

Concerning the fibrinolytic properties, it is well known that the normal balance between profibrinolytic and antifibrinolytic factors is altered in APL. Several events may contribute to an increased fibrinolysis. Secondary fibrinolysis may occur as a response to DIC at the onset of the disease. Leukemic promyelocytes contain both u-PA and t-PA.^59^–^61^ In addition, as previously described, APL blasts express increased levels of annexin II-associated fibrinolytic activity.^38^ Additional data suggest that retinoids induce a rapid increase of u-PA activity on APL cell surface, which is promptly down-regulated by an increased production of PA inhibitors, including PAI-1 and PAI-2.^32^ These mechanisms can contribute to a reduction of fibrinolytic activity in APL cells in response to ATRA. Recent data confirm that after induction therapy with either ATRA or combination of ATRA+ATO, the levels of FDP, D-dimer, plasminogen and fibrinogen normalize in 2–3 weeks, while the expression of Annexin II in APL blast cells is downregulated and the production of plasmin in APL cells is reduced.^55^ It has been hypothesized that part of the coagulopathy of APL is related to increased proteolysis by proteases, such as elastase, that degrade fibrinogen and other clotting factors.^62^–^63^ Increased plasma levels of elastase are indeed described in patients with acute leukemia.^62^,^64^ Elastase can degrade fibrinogen, producing a pattern of FDPs different from those produced by plasmin cleavage.^65^–^66^ However, in an *in vitro* study, freshly isolated APL blasts expressed lower fibrinolytic and proteolytic activities compared to mature neutrophils.^45^

The maintenance levels of coagulation inhibitors antithrombin (AT) and protein C (PC) may distinguish the coagulopathy of APL from typical DIC complicating other clinical conditions (e.g. sepsis). Although experimental DIC can occur in the presence of normal levels of AT, such findings are not typical in clinical practice. Of interest, however, is the observation by Rodeghiero and colleagues that reduced levels of AT and PC in patients with acute leukemia tend to occur in those patients with hepatic dysfunction. Patients with acute leukemia and DIC with normal liver function in their series usually had normal levels of the inhibitors.^67^

In line with these findings, the plasma elastase levels are elevated at the time of diagnosis of APL, most likely as the result of cell degranulation and lysis. However, ATRA therapy does not appear to affect these levels.^25^ Furthermore, no relation has been observed between plasma elastase concentration and the levels of D-dimer or other hemostatic variables during treatment with ATRA. These data, together with the data of De Stefano et al.,^45^ cast doubt on the earlier hypothesis that elastase makes an important contribution to the bleeding disorder of patients with APL or other myeloid leukemias.^64^

### 2. Procoagulant microparticles

Recently, considerable attention has been paid to TF circulating in blood in association with sub-cellular membrane vesicles, so-called plasma microparticles (MPs). Elevated levels of TF-positive MPs have been reported in acute leukemias.^68^

MPs are cell-derived membrane fragments measuring 0.1–1.0 mm, originating from normal cells, such as platelets, blood cells and endothelial cells (EC), or malignant cells. Soluble or free TF found in the plasma is carried by MPs and binds directly to factor VIIa.^69^ TF-MPs also facilitate the binding of cells and platelets to neutrophils and monocytes via P-selectin.^50^ Thus, hypothetically, MPs are involved directly and indirectly in activating coagulation. In APL, the leukemic promyelocytes express a high level of TF, and, as a consequence the MPs derived from the leukemic cells also carry TF. Thus the populations of TF-bearing MPs in APL plasma are correspondingly increased. In APL patients, at onset of disease, circulating MPs are mostly from the APL promyelocytes. During ATRA treatment, the population of promyelocytes decreases and the platelet counts return to normal and therefore the number of MPs decreases.^70^ Other relevant MPs-bound hemostatic proteins, such as tPA, PAI-1 and annexin II, have been found in the plasma of APL patients. However, more studies are needed to verify MPs significance as a prothrombotic factor in APL.

### 3. Cytokine release

Leukemic cells secrete various cytokines, including interleukin-1b (IL-1beta) and tumor necrosis factor (TNF-alpha).^71^ An increased secretion of IL-1beta has been observed in leukemic promyelocytes from patients with DIC compared to patients without DIC.^72^ Both TNF-alpha and IL-1beta induce the expression of TF and PAI-1 by EC and down-regulate the expression of EC thrombomodulin (TM). TM is a membrane receptor of vascular EC with a potent anticoagulant function.^73^ It binds and forms a complex with thrombin to activate the natural anticoagulant Protein C. Upregulation of the procoagulant TF with downregulation of the anticoagulant TM/Protein C system converts the normal anticoagulant endothelium into a prothrombotic endothelium. ATRA up-regulates the ability of leukemic cells to produce cytokines. This effect should shift the balance at the endothelium to the prothrombotic side; however, ATRA also appears to protect the endothelium *in vitro* against the prothrombotic assault of inflammatory cytokines. ATRA prevents both the down-regulation of TM and the up-regulation of TF induced by TNF-alpha^48^ and by IL-1beta produced by NB4 promyelocytic cells.^49^ Therefore, although ATRA increases cytokine synthesis by APL cells, it also appears to protect the endothelium against the prothrombotic stimulus of these mediators through a complex set of interactions.

### 4. Adhesion molecules

The expression on the surface of tumor cells of adhesion molecules and/or their counter-receptors permits the direct interaction of these cells with the host cells, including EC, platelets and leukocytes. The attachment of leukemic cells to vascular EC is relevant to promote localized clotting activation to the vessel wall and to start the microthrombi formation.

Activation of the endothelium by IL-1beta or TNF-alpha also leads to an increase in the expression of EC surface adhesion molecules,^74^ such as ICAM-1 or VCAM-1, which among other functions, serve as the counter-receptors for leukemic cell membrane adhesion molecules (i.e. integrins, such as LFA-1 and Mac-1). Some cytokines mediate tumor cell adhesion to the endothelium and to the subendothelial matrix.^75^–^76^ Attachment of leukemic cells to the vessel wall via these adhesion molecules (with special emphasis on so-called junctional adhesion molecules or JAM), with subsequent trans-endothelial migration represents one potential mechanism to explain the higher incidence of vascular complications in acute leukemia in association with high white blood cell (WBC) counts. Indeed, both early mortality and the so-called retinoic acid syndrome (RAS), which is characterized by unexplained fever, weight gain, respiratory distress, interstitial pulmonary infiltrates, pleural and pericardial effusions, episodic hypotension and acute renal failure, have been correlated with the *de novo* WBC count, as well as the expression of one or more adhesion molecules and/or cytokines that promote cell–cell interaction.^77^–^79^ Both clinical and experimental evidence, therefore, supports the concept that in patients with high WBC counts, leukemic cells (particularly APL cells) promote both localized clotting activation and WBC aggregation by adhesive interactions and subsequent activation of ECs.^80^–^81^

Although ATRA increases the adhesion capacity of APL cells to the endothelium in vitro,^76^ pre-treatment of ECs with ATRA reverses this effect and actually results in impaired adhesion of APL cells to ECs. This anti-adhesive effect may be explained by the down-regulation of EC surface-specific counter-receptors by ATRA.^76^ Perhaps ATRA is unable to exert this same protective effect on the specialized endothelium of the lung, thus explaining the unusual features of the RAS. It seems likely that a further understanding of the pathogenesis of the RAS and its prevention, as well as better strategies for the treatment of the consumptive coagulopathy of APL, will evolve from an improved understanding of the biological properties of the fusion proteins of RAR-alpha.^82^

## Treatment of the Coagulopathy of APL

**Figure 3:**
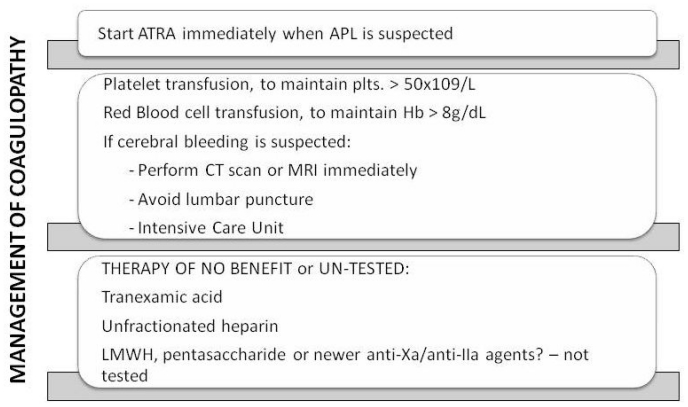
Schematics of the current approaches to the APL coagulopathy

Modern recommendations indicate that three simultaneous actions must be immediately undertaken when a diagnosis of APL is suspected: (1) the start of ATRA therapy; (2) the administration of supportive care with plasma and platelet transfusions; (3) the confirmation of genetic diagnosis.^83^–^85^ The mainstay of treatment of the coagulopathy of APL are shown in figure 3.

The main strategy in the management of the coagulopathy is early initiation of ATRA. This results in prompt resolution of the bleeding tendency and rapid normalization of coagulation tests and fibrinogen. Aggressive attention to the early initiation of supportive measures is particularly important in the management of acute leukemia, because effective chemotherapy often exacerbates the DIC and accentuates the bleeding syndrome by worsening the thrombocytopenia. The most important supportive tool, therefore, is the judicious use of platelet transfusion. The use of anticoagulants and antifibrinolytic agents, on the other hand, remains a hotly debated issue. The advent of ATRA treatment has ushered in a new era in the management of the coagulopathy of APL. Actually, because hemorrhagic complications can be a cause of death not only early during induction therapy but also before the diagnosis of APL, the current standard of care consists in the immediate start of ATRA even before the diagnosis of APL is confirmed at the genetic level. The simultaneous administration of ATRA and anthracycline-based chemotherapy is currently considered the standard induction treatment in newly diagnosed patients with APL; while the use of ATO-based regimens is under investigation and actually restricted to patients included in clinical trials or for those in whom chemotherapy is contraindicated.^9^,^86^ The combination therapy of ATRA+ATO is effective in inducing remission in newly diagnosed patients and may provide an alternative to ATRA+chemotherapy in this disease.^9^

## Platelet Transfusions, Heparin and Antifibrinolytic Agents

Consumption of platelets and coagulation factors causes bleeding symptoms, which interfere with therapy and may lead to hemorrhagic death. Platelet transfusions represent an essential part of the modern supportive care for all patients with acute leukemia, including APL patients. Prophylactic transfusion of platelets has resulted in a significant decrease in the incidence of fatal bleeding. In patients with APL, the bleeding risk and platelet transfusional requirements remain also high in the retinoic acid era. Current recommendations for patients with APL suggest that platelets should be transfused to maintain the platelet count above 20 × 10^9^/L in patients not actively bleeding and above 50 × 10^9^/L in those with active bleeding.^8^,^87^ Yanada ed al. observed that although most patients who developed severe hemorrhage were receiving frequent transfusions, the targeted levels of platelet count (>30 × 10^9^/L) and plasma fibrinogen (>150 mg/dL) were reached at the day of bleeding in only 71% and 40%, respectively suggesting that for patients at high risk of hemorrhage, more intensive transfusion may be beneficial.^88^

The role of heparin in the treatment of the coagulopathy complicating acute leukemia, especially APL, remains uncertain. Older studies, which used unfractionated heparin (UH), were small, retrospective, and uncontrolled. The benefit of UH therapy has never been proven in a prospective randomized trial. In a retrospective analysis of 268 patients with acute leukemia, no benefit was demonstrated of UH for the prevention of early hemorrhagic deaths, and no increase was observed in complete remission rate, or overall survival.^14^ To our knowledge no systematic studies have been reported of the use of LMWH or any of the newer anticoagulants to treat the THS of APL. A RCT utilizing either a LMWH or fondaparinux (pentasaccharide) in an effort to reduce the remaining death rate in APL due to the THS^89^–^90^ would seem appropriate. Extrapolating from data obtained from patients with ET or PV,^91^ it would also not be unreasonable to test the hypothesis that the anti-adhesive properties of LMWHs, which have been observed to reduce the interaction of solid tumor cells with the endothelium *in vitro*, might prevent some of the manifestations of the RAS in APL patients with high white blood cell counts.^90^

The use of antifibrinolytic agents such as epsilon-aminocaproic acid (EACA, Amicarw) or tranexamic acid, and/or protease inhibitors, such as Aprotinin (Trasylol) in the management of APL patients with bleeding have been considered, based on a few small studies,^92^–^93^ but no data from large-scale RCTs has been published. It is worth noting that an apparent increase in TE events occurred when antifibrinolytic agents were administered in conjunction with ATRA therapy in two studies.^94^ As mentioned above, in the PETHEMA group studies (e.g. LPA99) (32), tranexamic acid (100mg/kg/d) administered by continuous intravenous infusion until the platelet count was > 50×10^9^/L, as in their previous studies, “failed to demonstrate any impact on haemorrhage-associated mortality”.^95^ However, none of these agents has been tested in a RCT, where control subjects were not exposed to antifibrinolytic therapy.

However, the use of ATRA for remission induction has changed the natural history of APL and has helped to resolve the THS in most patients. While some of the mechanisms by which ATRA regulates the aberrant hemostatic system in patients with APL have been elucidated,^93^ the rate of early hemorrhagic deaths in APL (3–10%) has not changed significantly. Additional efforts to develop therapies that rapidly correct the coagulopathy are required, as noted above. New approaches using both anticoagulant and anti-inflammatory drugs should be considered. As the molecular basis for activation of clotting in hematologic malignancies becomes better elucidated, we anticipate the development of drugs that will target both the malignant process and the resultant THS.

## Conclusions

Coagulation laboratory abnormalities, indicating an activation of the hemostatic system, can be detected in virtually all patients with leukemias. The pathogenesis is complex and multifactorial. A prominent role is played by tumor-specific clot-promoting properties of leukemic cells themselves. In acute leukemia, bleeding manifestations prevail over localized thrombosis of large vessels. The risk of bleeding, due to thrombocytopenia and massive blood clotting activation with coagulation factors consumption, reaches a maximum in patients with APL. The coagulopathy of APL is characterized by low fibrinogen levels, prolongation of the PT and TT, and abnormal plasma levels of markers of hypecoagulation, hyperfibrinolysis and nonspecific proteolysis. Normal levels of AT and PC, coagulation inhibitors, in patients with the coagulopathy of APL cannot exclude DIC but may emphasize other features of the coagulopathy. This has raised some arguments against DIC, favoring the hypothesis of primary hyperfibrinolysis as the determinant of severe bleeding in acute leukemia. However, the nearly ubiquitous presence of elevated levels of fibrin D-dimer, and the increase of plasma levels of markers of clotting activation (F1+ 2, TAT and FPA), strongly favors the hypothesis of secondary or reactive hyperfibrinolysis occurring in response to activation of blood coagulation. Bleeding complications in patients with APL carry a high risk for mortality and, therefore, the use of prophylactic platelet transfusions is highly recommended. Although not discussed, aggressive management of infections is also very important, because viruses, Gram-negative and Gram-positive organisms can contribute to the development of DIC. In contrast, the routine use of anticoagulants and/or antifibrinolytic agents in the control or prevention of DIC cannot be recommended.

The advent of ATRA for induction and maintenance therapy of APL has profoundly modified the outlook for patients with this disease. Both ATRA and ATO have significantly improved and significantly reduced the early mortality rates from the management of bleeding in most series. These agents treat the underlying disease, therefore they fulfill the requirements for an optimal treatment of DIC in APL.

## References

[1] Falanga A, Marchetti M (2009). Venous thromboembolism in the hematologic malignancies. J Clin Oncol.

[2] Tallman MS, Kwaan HC (1992). Reassessing the hemostatic disorder associated with acute promyelocytic leukemia. Blood.

[3] Barbui T, FGaFA, Henderson ES (2002). Management of bleeding and thrombosis in acute leukaemia and chronic myeloproliferative disorders in Leukaemia. L.T.G.M.

[4] Barbui T, Finazzi G, Falanga A (1998). The impact of all-trans-retinoic acid on the coagulopathy of acute promyelocytic leukemia. Blood.

[5] Falanga A, Barbui T, Rickles FR (2008). Hypercoagulability and tissue factor gene upregulation in hematologic malignancies. Semin Thromb Hemost.

[6] Ku GH (2009). Venous thromboembolism in patients with acute leukemia: incidence, risk factors, and effect on survival. Blood.

[7] de la Serna J (2008). Causes and prognostic factors of remission induction failure in patients with acute promyelocytic leukemia treated with all-trans retinoic acid and idarubicin. Blood.

[8] Falanga A, Rickles FR (2003). Pathogenesis and management of the bleeding diathesis in acute promyelocytic leukaemia. Best Pract Res Clin Haematol.

[9] Sanz MA, Lo-Coco F (2011). Modern approaches to treating acute promyelocytic leukemia. J Clin Oncol.

[10] Warrell RP (1993). Acute promyelocytic leukemia. N Engl J Med.

[11] Golomb HM (1980). "Microgranular" acute promyelocytic leukemia: a distinct clinical, ultrastructural, and cytogenetic entity. Blood.

[12] Rovelli A (1992). Microgranular variant of acute promyelocytic leukemia in children. J Clin Oncol.

[13] Fenaux P (1993). Management of acute promyelocytic leukemia. Eur J Haematol.

[14] Rodeghiero F (1990). Early deaths and anti-hemorrhagic treatments in acute promyelocytic leukemia. A GIMEMA retrospective study in 268 consecutive patients. Blood.

[15] Castaigne S (1990). All-trans retinoic acid as a differentiation therapy for acute promyelocytic leukemia. I. Clinical results. Blood.

[16] Tallman MS (1993). New insights into the pathogenesis of coagulation dysfunction in acute promyelocytic leukemia. Leuk Lymphoma.

[17] Rickles FR (2007). Bleeding and thrombosis in acute leukemia: what does the future of therapy look like?. Thromb Res.

[18] Falanga A (1998). Mechanisms of hypercoagulation in malignancy and during chemotherapy. Haemostasis.

[19] Bauer KA, Rosenberg RD (1984). Thrombin generation in acute promyelocytic leukemia. Blood.

[20] Myers TJ (1981). Fibrinopeptide A in acute leukemia: relationship of activation of blood coagulation to disease activity. Blood.

[21] Booth NA, Bennett B (1984). Plasmin-alpha 2-antiplasmin complexes in bleeding disorders characterized by primary or secondary fibrinolysis. Br J Haematol.

[22] Reddy VB (1990). Global and molecular hemostatic markers in acute myeloid leukemia. Am J Clin Pathol.

[23] Speiser W (1990). Hemostatic and fibrinolytic parameters in patients with acute myeloid leukemia: activation of blood coagulation, fibrinolysis and unspecific proteolysis. Blut.

[24] Falanga A (1995). Loss of blast cell procoagulant activity and improvement of hemostatic variables in patients with acute promyelocytic leukemia administered all-trans-retinoic acid. Blood.

[25] Dombret H (1993). Coagulation disorders associated with acute promyelocytic leukemia: corrective effect of all-trans retinoic acid treatment. Leukemia.

[26] Dombret H (1995). In vivo thrombin and plasmin activities in patients with acute promyelocytic leukemia (APL): effect of all-trans retinoic acid (ATRA) therapy. Leukemia.

[27] Kawai Y (1994). Rapid improvement of coagulopathy by all-trans retinoic acid in acute promyelocytic leukemia. Am J Hematol.

[28] Watanabe R (1997). Long-term follow-up of hemostatic molecular markers during remission induction therapy with all-trans retinoic acid for acute promyelocytic leukemia. Keio Hematology-Oncology Cooperative Study Group (KHOCS). Thromb Haemost.

[29] Avvisati G (1988). Acquired alpha-2-antiplasmin deficiency in acute promyelocytic leukaemia. Br J Haematol.

[30] Schwartz BS (1986). Epsilon-aminocaproic acid in the treatment of patients with acute promyelocytic leukemia and acquired alpha-2-plasmin inhibitor deficiency. Ann Intern Med.

[31] Menell JS (1999). Annexin II and bleeding in acute promyelocytic leukemia. N Engl J Med.

[32] Tapiovaara H (1994). Induction of differentiation of promyelocytic NB4 cells by retinoic acid is associated with rapid increase in urokinase activity subsequently downregulated by production of inhibitors. Blood.

[33] Federici AB (1996). Proteolysis of von Willebrand factor is decreased in acute promyelocytic leukaemia by treatment with all-trans-retinoic acid. Br J Haematol.

[34] Tallman MS (2002). All-trans retinoic acid in acute promyelocytic leukemia: long-term outcome and prognostic factor analysis from the North American Intergroup protocol. Blood.

[35] Boccaccio C (2005). The MET oncogene drives a genetic programme linking cancer to haemostasis. Nature.

[36] Rong Y (2005). PTEN and hypoxia regulate tissue factor expression and plasma coagulation by glioblastoma. Cancer Res.

[37] Yu JL (2005). Oncogenic events regulate tissue factor expression in colorectal cancer cells: implications for tumor progression and angiogenesis. Blood.

[38] Andoh K (1987). Tissue factor activity in leukemia cells. Special reference to disseminated intravascular coagulation. Cancer.

[39] Gouault Heilmann M (1975). The procoagulant factor of leukaemic promyelocytes: demonstration of immunologic cross reactivity with human brain tissue factor. Br J Haematol.

[40] Bauer KA (1989). Tissue factor gene expression in acute myeloblastic leukemia. Thromb Res.

[41] Hair GA (1996). Tissue factor expression in human leukemic cells. Leuk Res.

[42] Falanga A (1988). A new procoagulant in acute leukemia. Blood.

[43] Falanga A (1994). Cancer procoagulant in the human promyelocytic cell line NB4 and its modulation by all-trans-retinoic acid. Leukemia.

[44] Koyama T (1994). All-trans retinoic acid upregulates thrombomodulin and downregulates tissue-factor expression in acute promyelocytic leukemia cells: distinct expression of thrombomodulin and tissue factor in human leukemic cells. Blood.

[45] De Stefano V (1995). Effect of all-trans retinoic acid on procoagulant and fibrinolytic activities of cultured blast cells from patients with acute promyelocytic leukemia. Blood.

[46] Falanga A (1998). Cancer procoagulant and tissue factor are differently modulated by all-trans-retinoic acid in acute promyelocytic leukemia cells. Blood.

[47] Saito T (1996). Anticoagulant effects of retinoic acids on leukemia cells. Blood.

[48] Ishii H (1992). Retinoic acid counteracts both the downregulation of thrombomodulin and the induction of tissue factor in cultured human endothelial cells exposed to tumor necrosis factor. Blood.

[49] Falanga A (1996). All-trans-retinoic acid counteracts endothelial cell procoagulant activity induced by a human promyelocytic leukemia-derived cell line (NB4). Blood.

[50] Oeth P (1998). Retinoic acid selectively inhibits lipopolysaccharide induction of tissue factor gene expression in human monocytes. Blood.

[51] Zhu J (1999). Tissue factors on acute promyelocytic leukemia and endothelial cells are differently regulated by retinoic acid, arsenic trioxide and chemotherapeutic agents. Leukemia.

[52] Raelson JV (1996). The PML/RAR-alpha oncoprotein is a direct molecular target of retinoic acid in acute promyelocytic leukemia cells. Blood.

[53] Brand K (1991). Tissue factor mRNA in THP-1 monocytic cells is regulated at both transcriptional and posttranscriptional levels in response to lipopolysaccharide. Mol Cell Biol.

[54] Cheng GX (1999). Distinct leukemia phenotypes in transgenic mice and different corepressor interactions generated by promyelocytic leukemia variant fusion genes PLZF-RARalpha and NPM-RARalpha. Proc Natl Acad Sci U S A.

[55] Liu Y (2011). The expression of annexin II and its role in the fibrinolytic activity in acute promyelocytic leukemia. Leuk Res.

[56] Emadi A, Gore SD (2010). Arsenic trioxide - An old drug rediscovered. Blood Rev.

[57] Zhou J (2010). Phosphatidylserine exposure and procoagulant activity in acute promyelocytic leukemia. J Thromb Haemost.

[58] Zhang X (2001). The impact of arsenic trioxide or all-trans retinoic acid treatment on coagulopathy in acute promyelocytic leukemia. Zhonghua Nei Ke Za Zhi.

[59] Bennett B (1989). The bleeding disorder in acute promyelocytic leukaemia: fibrinolysis due to u-PA rather than defibrination. Br J Haematol.

[60] Francis RB, Seyfert U (1987). Tissue plasminogen activator antigen and activity in disseminated intravascular coagulation: clinicopathologic correlations. J Lab Clin Med.

[61] Stephens R (1988). Production of an active urokinase by leukemia cells: a novel distinction from cell lines of solid tumors. Leuk Res.

[62] Nevo S (1998). Acute bleeding after bone marrow transplantation (BMT)-incidence and effect on survival. A quantitative analysis in 1,402 patients. Blood.

[63] Brower MS, Harpel PC (1982). Proteolytic cleavage and inactivation of alpha 2-plasmin inhibitor and C1 inactivator by human polymorphonuclear leukocyte elastase. J Biol Chem.

[64] Egbring R (1977). Demonstration of granulocytic proteases in plasma of patients with acute leukemia and septicemia with coagulation defects. Blood.

[65] Sterrenberg L (1984). Anticoagulant properties of purified X-like fragments of human fibrinogen produced by degradation with leukocyte elastase. Thromb Haemost.

[66] Sterrenberg L, Nieuwenhuizen W, Hermans J (1983). Purification and partial characterization of a D-like fragment from human fibrinogen, produced by human leukocyte elastase. Biochim Biophys Acta.

[67] Rodeghiero F (1984). Liver dysfunction rather than intravascular coagulation as the main cause of low protein C and antithrombin III in acute leukemia. Blood.

[68] Van Aalderen MC (2011). Procoagulant myeloblast-derived microparticles in AML patients: changes in numbers and thrombin generation potential during chemotherapy. J Thromb Haemost.

[69] Conese M (1991). Inhibitory effect of retinoids on the generation of procoagulant activity by blood mononuclear phagocytes. Thromb Haemost.

[70] Kwaan HC, Rego EM (2010). Role of microparticles in the hemostatic dysfunction in acute promyelocytic leukemia. Semin Thromb Hemost.

[71] Griffin JD (1987). Secretion of interleukin-1 by acute myeloblastic leukemia cells in vitro induces endothelial cells to secrete colony stimulating factors. Blood.

[72] Cozzolino F (1988). Potential role of interleukin-1 as the trigger for diffuse intravascular coagulation in acute nonlymphoblastic leukemia. Am J Med.

[73] Van de Wouwer M, Collen D, Conway EM (2004). Thrombomodulin-protein C-EPCR system: integrated to regulate coagulation and inflammation. Arterioscler Thromb Vasc Biol.

[74] Mantovani A, Bussolino F, Dejana E (1992). Cytokine regulation of endothelial cell function. FASEB J.

[75] Giavazzi R (1993). Rolling and adhesion of human tumor cells on vascular endothelium under physiological flow conditions. J Clin Invest.

[76] Marchetti M (1996). All-trans-retinoic acid increases adhesion to endothelium of the human promyelocytic leukaemia cell line NB4. Br J Haematol.

[77] Di Noto R (1994). All-trans retinoic acid promotes a differential regulation of adhesion molecules on acute myeloid leukaemia blast cells. Br J Haematol.

[78] Larson RS, Brown DC, Sklar LA (1997). Retinoic acid induces aggregation of the acute promyelocytic leukemia cell line NB-4 by utilization of LFA-1 and ICAM-2. Blood.

[79] Vahdat L (1994). Early mortality and the retinoic acid syndrome in acute promyelocytic leukemia: impact of leukocytosis, low-dose chemotherapy, PMN/RAR-alpha isoform, and CD13 expression in patients treated with all-trans retinoic acid. Blood.

[80] Dubois C (1994). Modulation of IL-8, IL-1 beta, and G-CSF secretion by all-trans retinoic acid in acute promyelocytic leukemia. Leukemia.

[81] Marchetti C (1997). Endothelin and nitric oxide synthase in lymphatic endothelial cells: immunolocalization in vivo and in vitro. Anat Rec.

[82] Melnick A, Licht JD (1999). Deconstructing a disease: RARalpha, its fusion partners, and their roles in the pathogenesis of acute promyelocytic leukemia. Blood.

[83] Breccia M (2010). Early hemorrhagic death before starting therapy in acute promyelocytic leukemia: association with high WBC count, late diagnosis and delayed treatment initiation. Haematologica.

[84] Lo-Coco F (2010). Front-line treatment of acute promyelocytic leukemia with AIDA induction followed by risk-adapted consolidation for adults younger than 61 years: results of the AIDA-2000 trial of the GIMEMA Group. Blood.

[85] Tallman MS (2010). Updates on the treatment of acute promyelocytic leukemia. Clin Adv Hematol Oncol.

[86] Sanz MA (2008). Risk-adapted treatment of acute promyelocytic leukemia with all-trans retinoic acid and anthracycline monochemotherapy: long-term outcome of the LPA 99 multicenter study by the PETHEMA Group. Blood.

[87] Arbuthnot C, Wilde JT (2006). Haemostatic problems in acute promyelocytic leukaemia. Blood Rev.

[88] Yanada M (2007). Severe hemorrhagic complications during remission induction therapy for acute promyelocytic leukemia: incidence, risk factors, and influence on outcome. Eur J Haematol.

[89] Blom JW (2005). Malignancies, prothrombotic mutations, and the risk of venous thrombosis. JAMA.

[90] Falanga A (2005). Pathogenesis of thrombosis in essential thrombocythemia and polycythemia vera: the role of neutrophils. Semin Hematol.

[91] Falanga A (2005). Leukocyte-platelet interaction in patients with essential thrombocythemia and polycythemia vera. Exp Hematol.

[92] Barbui T, Finazzi G, Falanga A, Henderson ES, Graves MF (2001). The management of bleeding and thrombosis in acute leukemia and chronic myeloproliferative disorders. Leukemia.

[93] Falanga A, Donati MB (2001). Pathogenesis of thrombosis in patients with malignancy. Int J Hematol.

[94] Hashimoto S (1994). Fatal thromboembolism in acute promyelocytic leukemia during all-trans retinoic acid therapy combined with antifibrinolytic therapy for prophylaxis of hemorrhage. Leukemia.

[95] Sanz MA (2004). Risk-adapted treatment of acute promyelocytic leukemia with all-trans-retinoic acid and anthracycline monochemotherapy: a multicenter study by the PETHEMA group. Blood.

